# ES‐62 Protects Against Collagen‐Induced Arthritis by Resetting Interleukin‐22 Toward Resolution of Inflammation in the Joints

**DOI:** 10.1002/art.38392

**Published:** 2014-05-27

**Authors:** Miguel A. Pineda, David T. Rodgers, Lamyaa Al‐Riyami, William Harnett, Margaret M. Harnett

**Affiliations:** ^1^ University of GlasgowGlasgowUK; ^2^ University of StrathclydeGlasgowUK

## Abstract

**Objective:**

The parasitic worm–derived immunomodulator ES‐62 protects against disease in the mouse collagen‐induced arthritis (CIA) model of rheumatoid arthritis (RA) by suppressing pathogenic interleukin‐17 (IL‐17) responses. The Th17‐associated cytokine IL‐22 also appears to have a pathogenic role in autoimmune arthritis, particularly in promoting proinflammatory responses by synovial fibroblasts and osteoclastogenesis. The present study was undertaken to investigate whether the protection against joint damage afforded by ES‐62 also reflects suppression of IL‐22.

**Methods:**

The role(s) of IL‐22 was assessed by investigating the effects of neutralizing anti–IL‐22 antibodies and recombinant IL‐22 (rIL‐22) on proinflammatory cytokine production, synovial fibroblast responses, and joint damage in mice with CIA in the presence or absence of ES‐62.

**Results:**

Neutralization of IL‐22 during the initiation phase abrogated CIA, while administration of rIL‐22 enhanced synovial fibroblast responses and exacerbated joint pathology. In contrast, after disease onset anti–IL‐22 did not suppress progression, whereas administration of rIL‐22 promoted resolution of inflammation. Consistent with these late antiinflammatory effects, the protection afforded by ES‐62 was associated with elevated levels of IL‐22 in the serum and joints that reflected a desensitization of the synovial fibroblast responses. Moreover, neutralization of IL‐22 during the late effector stage of disease prevented ES‐62–mediated desensitization of synovial fibroblast responses and protection against CIA.

**Conclusion:**

IL‐22 plays a dual role in CIA, being pathogenic during the initiation phase while acting to resolve inflammation and joint damage during established disease. Harnessing of the tissue repair properties of IL‐22 by ES‐62 highlights the potential for joint‐targeted therapeutic modulation of synovial fibroblast responses and consequent protection against bone damage in RA.

Rheumatoid arthritis (RA) is a chronic autoimmune disorder characterized by synovial inflammation and resultant progressive joint damage. It has become increasingly evident that IL‐17–dependent responses play a central role in RA, with aberrant regulation of Th17 cells being implicated in disease onset and progression (1, 2). In particular, IL‐17 recruits neutrophils to the joint and induces secretion of proinflammatory cytokines by synovial fibroblasts, resulting in the promotion of osteoclastogenesis and hence, cartilage and bone destruction (3). Elevated numbers of Th17 cells have been found in patients with RA (4, 5), and a pathogenic role of IL‐17 in arthritis has been confirmed in animal models (6, 7). ES‐62, a phosphorylcholine (PC)–containing immunomodulator secreted by the filarial nematode *Acanthocheilonema viteae* (8), protects against collagen‐induced arthritis (CIA) in mice (9, 10) by down‐regulating IL‐17 responses, via targeting of an inflammatory cellular network involving dendritic cells, γ/δ T cells, and Th17 cells (11).

Th17 cells also secrete IL‐22, a cytokine generally considered to be proinflammatory because of its coexpression with IL‐17 during in vitro differentiation of Th17 cells (12). However, there is increasing evidence that IL‐17 and IL‐22 are differentially regulated and often produced in vivo by different lymphocyte subsets. Thus, transforming growth factor β is not required, and IL‐6 is sufficient, to induce IL‐22 production by T cells (13)—unlike the case for IL‐17. However, the transcription factor aryl hydrocarbon receptor is essential for the production of IL‐22 (14) by CCR10+ “Th22” cells that can be discriminated from Th17 cells (15). IL‐22 is also produced by innate lymphocytes (lymphoid tissue–inducer cells, γ/δ T cells, and natural killer cells) (16), but the widely expressed IL‐22 receptor (IL‐22R1–IL‐10Rβ) is not usually expressed by hemopoietic cells (17). Thus, IL‐22 appears to provide a link between the immune system and other tissues to promote their innate immunity, in particular, to enhance antimicrobial defense and tissue repair (17, 18). Reflecting these pleiotropic effects, IL‐22 has been reported to exhibit both protective effects (hepatitis and inflammatory bowel disease) and pathogenic effects (psoriasis) (13, 19, 20, 21) in inflammatory disease.

In the context of RA, mice that are deficient in IL‐22 are less susceptible to CIA and/or develop less severe disease (22, 23). Moreover, levels of IL‐22 and Th22 cells have been found to be elevated in the periphery and synovia of RA patients (24, 25, 26), and IL‐22 has been shown to induce proliferation of synovial fibroblasts and promote RANKL production and osteoclastogenesis in vitro (27). We therefore investigated whether the protective effects of ES‐62 were also associated with targeting of such IL‐22 responses. Surprisingly, these studies revealed that IL‐22 can play dual pathogenic and protective roles in CIA and that ES‐62 harnesses the cytokine's antiinflammatory effects on synovial fibroblasts, to mediate its protection against joint destruction. In describing a novel mechanism by which a parasitic helminth–derived product acts to reduce autoimmune arthritis, these findings contribute to our fundamental understanding of IL‐22 immunobiology and identify novel therapeutic targets in inflammatory disease.

## MATERIALS AND METHODS

### Mice

Animals were maintained in the Biological Services Units at the University of Glasgow and the University of Strathclyde, in accordance with Home Office UK Licenses PPL60/4300, PPL60/3791, PIL60/12183, PIL60/12950, and PIL60/9576 and the respective ethics review boards of these universities. CIA was induced in 8–10‐week‐old male DBA/1 mice (Harlan Olac) by intradermal immunization with bovine type II collagen (MD Biosciences) in Freund's complete adjuvant (day 0) and by intraperitoneal (IP) administration in phosphate buffered saline (PBS) (day 21). Purified endotoxin‐free ES‐62 (2 μg/dose) or PBS was administered subcutaneously on days −2, 0, and 21 (9), and cells were recovered from draining lymph nodes (DLNs) and joints as previously described (11). Mice were treated with endotoxin‐free recombinant IL‐22 (rIL‐22; ImmunoTools) (1 μg/dose IP or 0.25 μg/dose footpad injection, twice weekly as indicated) or endotoxin‐free mouse IgG (Europa Bioproducts) (100 μg/dose IP twice weekly beginning on day 7) or anti–IL‐22 antibodies (28) purified from the AM22.1 hybridoma (100 μg/dose IP twice weekly as indicated) (hybridoma kindly provided by Dr. J. C. Renauld, Ludwig Institute for Cancer Research, Brussels, Belgium). There were no significant differences in articular scores between PBS‐treated mice with CIA that were treated with mouse IgG and those that were not treated with mouse IgG. Mice were monitored for clinical symptoms of arthritis, which were scored as 0 (normal), 1 (erythema), 2 (erythema plus swelling), 3 (extension of swelling), or 4 (loss of function); the overall disease score was the sum of the scores for each of the 4 limbs. The date mice were removed from the study varied slightly as euthanasia is required when a score of 10 is reached or clinical symptoms develop in all 4 limbs. Other than dial caliper (Kroeplin) analysis of paw thickness, all analyses were also performed on the day the animal was removed from the study.

### Ex vivo analysis

DLN cells (10^6^/ml) were incubated with or without phorbol myristate acetate (PMA) (50 ng/ml)/ionomycin (500 ng/ml) (Sigma‐Aldrich) for 1 hour, followed by addition of 10 μg/ml brefeldin A (Sigma‐Aldrich) for 5 hours at 37°C with 5% CO_2_. Live cells were discriminated with Live/Dead Fixable Aqua Dye (Invitrogen), and phenotypic markers labeled using fluorescein isothiocyanate (FITC)–conjugated anti‐CD3 (BD PharMingen) and PerCP‐conjugated anti‐CD4 or biotinylated anti‐CD4 antibodies (detected with Alexa Fluor 450–conjugated streptavidin) (BD PharMingen) before the cells were fixed and permeabilized according to protocols recommended by the supplier (BioLegend). Cytokines were stained using phycoerythrin (PE)–Cy7–conjugated anti–interferon‐γ (anti‐IFNγ), allophycocyanin‐conjugated or PerCP–Cy5.5–conjugated anti–IL‐17A (BioLegend), or PE‐conjugated anti–IL‐22 antibodies (R&D Systems) for 30 minutes prior to analysis by flow cytometry, with gating according to appropriate isotype controls. Detection of biologically relevant IL‐22+ cells was validated by in vitro Th17/22 differentiation assays using 2 anti–IL‐22 antibodies (clone 140301 [R&D Systems] and clone 1H8PWSR [eBioscience]) with or without rIL‐22 pretreatment (10–40 μg/ml); essentially the same results were obtained with both clones when dead cells were excluded from the analyses. Cells were extracted from the joints of mice with CIA by collagenase digestion and incubated with 10 μg/ml brefeldin A for 5 hours at 37°C. Following live cell discrimination with Live/Dead Fixable Aqua Dye, cells were labeled with PerCP‐conjugated anti‐Gr1 and FITC‐conjugated anti‐CD11b antibodies (eBioscience) before being fixed and permeabilized for staining for IL‐17, IL‐22, and IFNγ expression.

### Cytokine measurement

Levels of IL‐17A, IL‐22, and IL‐6 were determined by enzyme‐linked immunosorbent assay, according to the recommendations of the manufacturers (BioLegend, R&D Systems, and eBioscience, respectively).

### Synovial fibroblast explant cultures

Single‐cell suspensions recovered from all 4 limbs by collagenase digestion (29) were pooled to avoid biasing results. Cells were cultured for 12 hours in Dulbecco's modified Eagle's medium (Invitrogen) containing 2 m*M* glutamine, 50 units/ml penicillin, 50 μg/ml streptomycin, and 10% fetal calf serum, after which nonadherent cells were removed. Adherent cells were cultured for 7 days and phenotyped using biotinylated anti‐CD54, PE–Cy7–conjugated anti‐CD106, and PerCP‐conjugated anti‐CD90.2 antibodies (eBioscience). Synovial fibroblasts were stimulated in vitro with murine rIL‐17 or rIL‐22. After 24 hours, supernatants were collected for analysis.

### Immunofluorescence

Preparation of tissue sections (7 μm), staining with hematoxylin and eosin, and detection of IL‐17 expression were performed as previously described (11). To detect IL‐22 expression, samples were stained for 12 hours at 4°C with a rat anti‐mouse IL‐22 antibody (rat IgG isotype control; R&D Systems) with DAPI counterstaining, followed by detection using a biotinylated goat anti‐rat IgG antibody and Alexa Fluor 647–conjugated streptavidin. Images were obtained using an LSM 510 Meta confocal laser coupled to an Axiovert 200 microscope (Zeiss) and analyzed with Zeiss LSM Image Browser software.

### Statistical analysis

The significance of differences was determined by Student's unpaired 1‐tailed *t*‐test or by one‐way analysis of variance followed by Newman‐Keuls post hoc test. Articular scores were assessed by Mann‐Whitney test. *P* values less than 0.05 were considered significant.

## RESULTS

### Differential regulation of IL‐17 and IL‐22 responses by ES‐62 in mice with CIA

**Figure 1:**
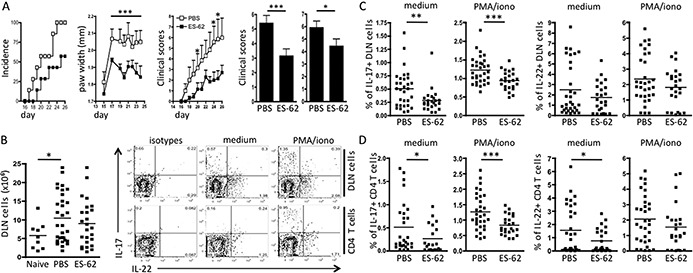
ES‐62 differentially targets interleukin‐17 (IL‐17)– and IL‐22–producing draining lymph node (DLN) cells in mice with collagen‐induced arthritis (CIA). **A,** Percent CIA incidence, mean ± SEM paw width, and mean ± SEM articular (clinical) score at various time points in a representative experiment (n = 7 phosphate buffered saline [PBS]–treated mice and 7 ES‐62–treated mice) (first through third panels), and mean ± SEM articular score (pooled from 6 independent experiments) at the time of removal from the experiment for all mice (fourth panel; n = 38 PBS‐treated mice and 36 ES‐62–treated mice) and mice that had developed CIA (fifth panel; n = 34 PBS‐treated mice and 24 ES‐62–treated mice). **B,** Number of DLN cells in naive mice (n = 9), PBS‐treated mice with CIA (n = 32), and ES‐62–treated mice with CIA (n = 25), and representative plots of intracellular IL‐22 and IL‐17 expression from experiments using medium‐treated or phorbol myristate acetate plus ionomycin (PMA/iono)–stimulated DLN cells and CD4+ T cells from PBS‐treated mice with CIA. **C** and **D,** Percentages of DLN cells (**C**) or CD4+ T cells (**D**) expressing IL‐17 or IL‐22 in PBS‐treated mice with CIA (n = 32) and ES‐62–treated mice with CIA (n = 24). In **B** (left panel), **C,** and **D,** each symbol represents an individual mouse; bars show the mean. ∗ = *P* < 0.05 (versus ES‐62 on the days indicated, in the second and third panels of **A**); ∗∗ = *P* < 0.01; ∗∗∗ = *P* < 0.001.

Compared to the PBS‐treated group, the incidence of CIA was significantly reduced in mice treated with ES‐62, as were the degree of hind paw swelling and clinical scores (even among ES‐62–treated animals that did develop CIA) (Figure 1A). In parallel with the clinical findings, the total number of DLN cells was significantly increased in mice with CIA treated with PBS, but not in those exposed to ES‐62 in vivo, compared to the number in naive mice without CIA (Figure 1B). However, while the proportions of DLN cells and CD4+ T cells that produced IL‐17 in response to ex vivo stimulation with either medium or PMA plus ionomycin were significantly reduced by exposure to ES‐62 in vivo, only the levels of unstimulated IL‐22–producing CD4+ T cells were significantly suppressed (Figures 1C and D). Moreover, whereas the ability of DLN and CD4+ cells to generate IL‐17 ex vivo was significantly increased by stimulation with PMA plus ionomycin (*P* < 0.01 for both), this was not the case with regard to production of IL‐22, as reflected by the finding that these cytokines were generated by distinct subsets of DLN and CD4+ cells (Figure 1B).

**Figure 2:**
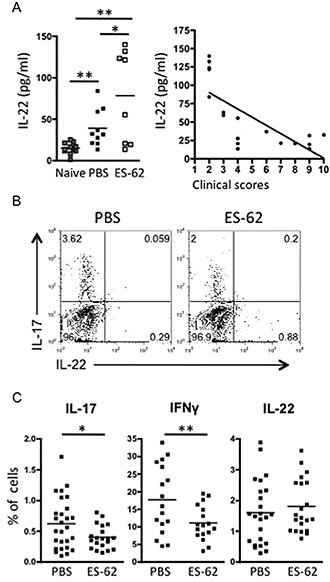
ES‐62–dependent up‐regulation of IL‐22 correlates with protection against CIA. **A,** Serum IL‐22 levels in naive mice (n = 8), PBS‐treated mice with CIA (n = 10), and ES‐62–treated mice with CIA (n = 8), and inverse correlation (Pearson's r = −0.7298, *P* < 0.0006) between serum IL‐22 levels and clinical scores in 18 PBS‐ or ES‐62–treated mice with CIA (each symbol represents the mean value from triplicate analyses in an individual mouse). **B,** Representative plots of intracellular IL‐17 and IL‐22 expression in pooled joint cells from PBS‐treated mice with CIA (n = 4) and ES‐62–treated mice with CIA (n = 4). **C,** Percentages of joint cells expressing IL‐17, interferon‐γ (IFNγ), and IL‐22 in PBS‐treated mice with CIA (n = 27, 17, and 24 for IL‐17, IFNγ, and IL‐22, respectively) and ES‐62–treated mice with CIA (n = 20, 17, and 21, respectively). In **A** (left panel) and **C,** each symbol represents an individual mouse; bars show the mean. ∗ = *P* < 0.05; ∗∗ = *P* < 0.01; ∗∗∗ = *P* < 0.001. See Figure 1 for other definitions.

Although no differences could be detected at early time points, in accordance with previous observations in human patients (30) and the proposed pathogenic role of IL‐22 in the CIA model (22, 23), the mean serum level of IL‐22 was increased in PBS‐treated mice with CIA compared to naive controls at the last assessment (Figure 2A). Rather unexpectedly, ES‐62–treated mice exhibited an even higher mean serum level of IL‐22. As this group segregated into high and low IL‐22 producers, we investigated whether this was related to disease progression and found that IL‐22 levels (in both PBS‐treated and ES‐62–treated mice) correlated inversely with CIA severity (Figure 2A). These findings are in direct contrast to the association of serum IL‐17 levels with articular score and their significant reduction by in vivo exposure to ES‐62 (11).

Analysis of joint‐infiltrating cells revealed that IL‐17 and IL‐22 were also produced predominantly by distinct cell subsets within this population, although it was not clear whether these included Th cells due to our failure to detect expression of CD4 (presumably due to its cleavage during the collagenase extraction procedure). Nevertheless, a reduced proportion of the cells expressed IL‐17, and an increased proportion generated IL‐22, in the mice with CIA that had been exposed to ES‐62 in vivo (Figure 2B). Independent analysis of individual mice showed that, whereas exposure to ES‐62 resulted in significant suppression of the levels of IL‐17– and also IFNγ‐producing cells, the levels of IL‐22–generating cells were maintained and even increased, although this did not reach statistical significance (Figure 2C).

Onset of arthritis was detected in a few mice prior to challenge with collagen. While exposure to ES‐62 reduced the incidence of this (11%, versus 23% in PBS‐treated mice), it did not significantly ameliorate pathology in the mice that developed disease. Perhaps consistent with this, ES‐62 did not suppress the (reduced) levels of IL‐17–producing cells in the joints of these mice. The differential IL‐17:IL‐22 ratios (0.49 and 0.21 in the PBS‐ and ES‐62–treated groups, respectively, postchallenge; 0.26 and 0.31, respectively, prechallenge) and the ES‐62 sensitivity observed in the 2 groups further supported the idea that resetting of the IL‐17/IL‐22 balance in the joint correlates with ES‐62–mediated protection and perhaps suggests that additional/alternative inflammatory parameters contributed to the pathogenesis in mice that developed arthritis prior to challenge.

### ES‐62–mediated protection against CIA is dependent on IL‐22.

**Figure 3:**
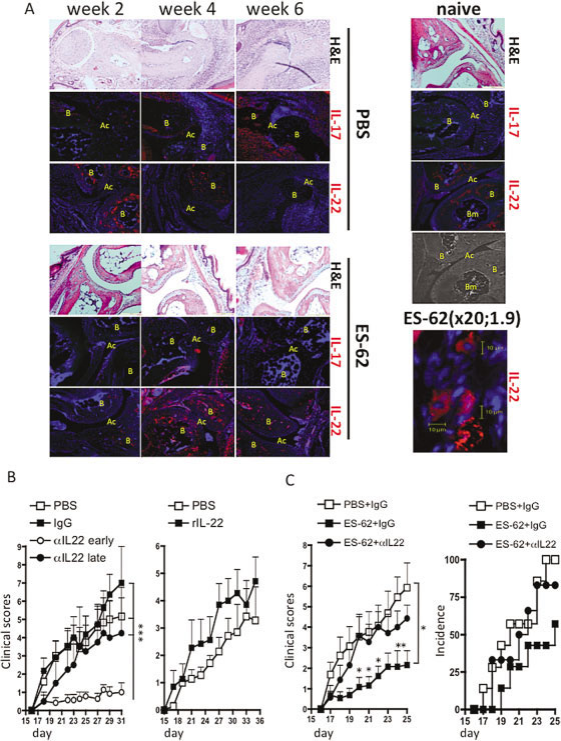
ES‐62 protection against CIA requires IL‐22. **A,** Joint sections from representative PBS‐treated mice with CIA, ES‐62–treated mice with CIA, and naive mice at weeks 2, 4, and 6 (articular scores 0, 4, and 8, respectively, at weeks 2, 4, and 6 in the PBS‐treated mice and 0, 2, and 2, respectively, in the ES‐62–treated mice). Sections were stained with hematoxylin and eosin (H&E) or for IL‐17 or IL‐22 (red) or nuclei (DAPI; blue). Isotype control sections were negative for IL‐17 and IL‐22. Joint structure is shown in the grayscale image. **B** = bone; **AC** = articular cavity; **BM** = bone marrow. Original magnification × 20 (1.9 zoom). **B,** Mean ± SEM articular scores in mice with CIA treated with PBS (n = 12), murine IgG (100 μg/dose; n = 11), or anti–IL‐22 (100 μg/dose) twice weekly from day 7 (early αIL‐22; n = 14) or from day 19 (late αIL‐22; n = 4), or treated intraperitoneally (IP) with PBS (n = 7) or recombinant IL‐22 (rIL‐22) (1 μg/dose; n = 7) twice weekly from day 7. ∗∗∗ = *P* < 0.001. **C,** Mean ± SEM articular scores in mice with CIA treated with PBS plus IgG (n = 13), ES‐62 plus IgG (n = 13), or ES‐62 plus anti–IL‐22 (n = 12) and with antibodies administered IP twice weekly from day 19 onward (100 μg/dose) and ES‐62 administered on days −2, 0, and 21 (2 μg/dose), and percent incidence of disease (score >1) by treatment group. ∗ = *P* < 0.05 (*P* values shown for specific days are versus treatment with ES‐62 plus anti–IL‐22). See Figure 1 for other definitions.

In studies of the relationship of IL‐17 and IL‐22 expression with joint pathology in situ, we have shown that, while expression of IL‐17 was essentially absent in the joints of naive mice, increasing levels were observed throughout the progression of CIA and correlated with induction of joint pathology (11). In the present study we observed that in vivo exposure to ES‐62 suppressed both the expression of IL‐17 in the joints and the development of joint disease (Figure 3A). In contrast, IL‐22 was expressed in the joints of naive mice predominantly in the bone area (not bone marrow), but some IL‐22+ cells (∼10 μm) could also be detected in the synovium. Such IL‐22 expression was also evident in mice with CIA, but following an increase (within 2 weeks) during the disease initiation phase, it subsequently decreased over time. However, at 4 weeks some IL‐22+ cells were found around the periphery of the bone in mice with CIA, but not in naive or ES‐62–treated mice.

Exposure to ES‐62 in vivo induced an inverse pattern of expression, with the parasite product suppressing the early peak of IL‐22 expression (Figure 3A), perhaps mirroring its inhibition of Th22 responses observed in the DLNs, but inducing strong expression at later time points consistent with its induction of IL‐22+ joint cells (peaking at week 4). These include synovium cells (∼20 μm; 20× digital magnification, 1.9 zoom) not seen in PBS‐treated mice with CIA. In terms of their bipolar, spindle shape and prominent secretory machinery (31) as evidenced by punctate IL‐22 staining, these cells are reminiscent of the IL‐22–producing fibroblast‐like synoviocytes recently reported to be protective against RA (32); hence, our results support the notion that ES‐62 mediates protection against joint pathology via an IL‐22–dependent mechanism.

To investigate the pathogenic role of IL‐22, mice were treated during the initiation phase (twice weekly from day 7 of disease, as preliminary experiments established that levels of IL‐22–producing DLN cells were elevated within 7–14 days) with either neutralizing anti–IL‐22 antibodies or rIL‐22 to determine whether these reagents could, respectively, block or promote development of CIA (Figure 3B). Exposure to neutralizing anti–IL‐22 antibodies essentially abrogated development of CIA, with no similar effect obtained with the use of irrelevant IgG. Administration of rIL‐22 tended to promote both disease onset and increased severity (Figure 3B); indeed, the number of limbs affected in the rIL‐22–treated cohort necessitated termination of these experiments before full pathology was established in the PBS group. In contrast, when neutralizing anti–IL‐22 antibodies were not administered until around the time of onset of pathology but prior to challenge with collagen (day 19), there was no significant disruption of the development of CIA (Figure 3B), supporting the notion that IL‐22 has a pathogenic role during the early, but not the later, phase of CIA.

As ES‐62 acted to maintain and/or enhance IL‐22 levels in serum, DLN cells, and joints during established disease (within 3–4 weeks), we also investigated whether administration of neutralizing anti–IL‐22 antibodies around the time of onset of joint pathology (day 19) would abrogate the protective effects of ES‐62. The results of these experiments (Figure 3C) indicated that the protective effects of ES‐62 were indeed dependent on IL‐22.

### ES‐62 and IL‐22 down‐regulate synovial fibroblast responses and suppress joint inflammation

**Figure 4:**
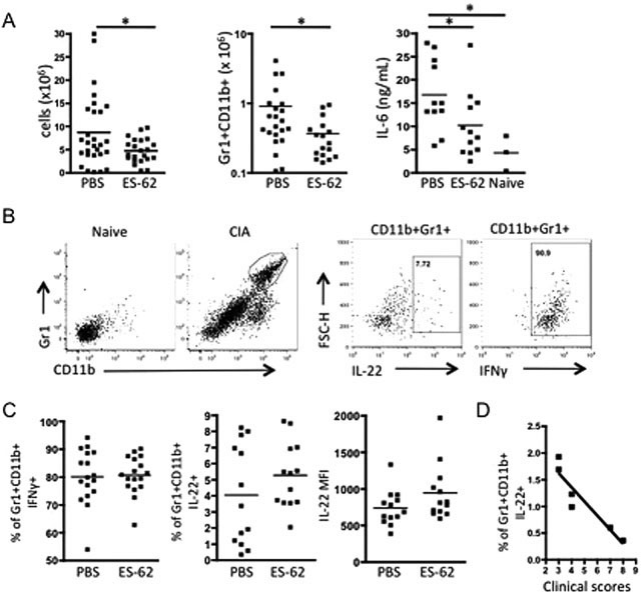
ES‐62 modulates cellular infiltration of the joints of mice with CIA. **A,** Numbers of infiltrating cells in the joints of PBS‐treated mice with CIA (n = 28) and ES‐62–treated mice with CIA (n = 22), numbers of CD11b+Gr1+ cells in the joints of PBS‐treated mice with CIA (n = 22) and ES‐62–treated mice with CIA (n = 17), and production of IL‐6, determined by enzyme‐linked immunosorbent assay, in joint cells (10^6^/ml) from PBS‐treated mice with CIA (n = 11), ES‐62–treated mice with CIA (n = 12), and naive mice (n = 3) (each symbol represents the mean value from triplicate analyses in an individual mouse). **B,** Gating strategy for analysis of Gr1+CD11b+ cells isolated from the joints of PBS‐treated mice with CIA, and their expression of IL‐22 and interferon‐γ (IFNγ). **C,** Percentages of IFNγ+ and IL‐22+ CD11b+Gr1+ joint cells, and mean fluorescence intensity (MFI) of IL‐22 expression by CD11b+Gr1+ joint cells from PBS‐treated mice with CIA (n = 17 and 13 for IFNγ and IL‐22, respectively) and ES‐62–treated mice with CIA (n = 17 and 13, respectively). **D,** Inverse correlation (Pearson's r = −0.86, *P* = 0.0075) between the percentage of IL‐22+CD11b+Gr1+ cells and clinical scores in mice with CIA. (The same trend was found in 2 additional independent experiments.) In **A** and **C,** each symbol represents an individual mouse; bars show the mean. ∗ = *P* < 0.05. See Figure 1 for other definitions.

To investigate the mechanisms involved in the observed protection against CIA, we examined the effects of IL‐17, IL‐22, and ES‐62 on joint inflammation. Consistent with the suppression of pathogenic IL‐17 responses, the total number of infiltrating cells, and in particular, CD11b+Gr1+ neutrophils (Figure 4A), was significantly higher in the joints of mice with CIA treated with PBS relative to those exposed to ES‐62. Moreover, infiltrating cells from the ES‐62–treated mice showed significantly reduced levels of IL‐6 release (Figure 4A).

Most of the neutrophils (>70%) isolated from the joints of animals with CIA were able to produce IFNγ (Figure 4B), and while this proportion was not altered by exposure to ES‐62 (Figure 4C), their lower numbers would contribute to the observed reduction of IFNγ‐producing cells in the joint (Figure 2C). Some of the neutrophils appeared to express IL‐22 (Figure 4B), and the proportion of IL‐22+Gr1+CD11b+ cells and their levels of IL‐22 expression tended to be increased by ES‐62 (Figure 4C), although neither increase reached statistical significance. Nevertheless, such immunomodulation results in a shift in the balance of cytokines, with a relative reduction of IL‐6/IL‐17/IFNγ expression and increase in IL‐22 expression (Figures 2C, 4A, and 4C). Consistent with the ES‐62–mediated promotion of IL‐22 levels, there was a negative correlation between the proportion of this IL‐22+Gr1+CD11b+ subset of neutrophils and the severity of disease (Figure 4D), suggesting that ES‐62 promotes induction of an IL‐22+ neutrophil subset that infiltrates the joint to mediate protection against CIA.

**Figure 5:**
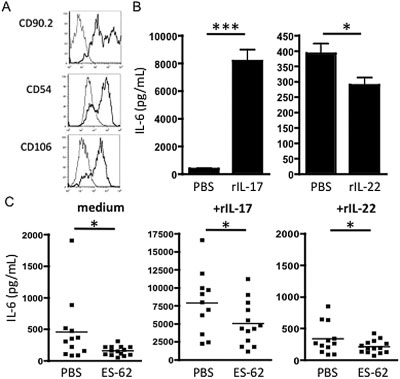
ES‐62 desensitizes IL‐17–mediated production of IL‐6 by synovial fibroblasts. **A,** CD90.2, CD54, and CD106 expression (black lines) by synovial fibroblasts from mice with CIA (gray lines represent isotype controls). Fibroblasts were cultured ex vivo for 7 days prior to in vitro stimulation. **B,** IL‐6 production by synovial fibroblasts from PBS‐treated mice with CIA, in response to recombinant IL‐17 (rIL‐17) or rIL‐22 (10 ng/ml). Three independent cultures of cells obtained by pooling mice (n = 7) within each group were performed. Values are the mean ± SEM (calculated using the mean result from the triplicate experiments). **C,** IL‐6 production over 24 hours by synovial fibroblast explant cultures (10^6^/ml) from individual PBS‐treated mice with CIA or ES‐62–treated mice with CIA, in response to fresh medium, rIL‐17 (10 ng/ml), or rIL‐22 (10 ng/ml). For PBS‐treated mice, n = 12, 11, and 12 for fresh medium, rIL‐17, and rIL‐22, respectively; for ES‐62–treated mice, n = 14, 13, and 14, respectively. Each symbol represents the mean value from triplicate determinations (by enzyme‐linked immunosorbent assay) in an individual mouse; bars show the mean. ∗ = *P* < 0.05; ∗∗∗ = *P* < 0.001. See Figure 1 for other definitions.

As hemopoietic cells generally do not express IL‐22R, we next investigated whether the IL‐22–dependent protective effects of ES‐62 reflected modulation of the function of synovial fibroblasts, which not only secrete cytokines (such as IL‐6) that recruit inflammatory cells (such as neutrophils) to the joint, but also act to directly mediate joint destruction by releasing proteolytic enzymes (matrix metalloproteinases) that degrade cartilage and by secreting factors (e.g., RANKL) that further contribute to bone destruction by activating osteoclastogenesis and promoting bone resorption (33, 34, 35). Consistent with this proposal, when explant cultures of synovial fibroblasts (CD90.2+CD54+CD106+) (Figure 5A) extracted from mice with CIA were incubated with rIL‐22 the production of IL‐6, rather than being stimulated, was inhibited to below basal levels (Figure 5B), in direct contrast with the results obtained with rIL‐17 incubation. Moreover, explant cultures of synovial fibroblasts from mice with CIA exposed to ES‐62 showed significantly reduced basal production of IL‐6 and were less responsive to rIL‐17 and rIL‐22 than fibroblast cultures from PBS‐treated mice (Figure 5C). Indeed, even though culture of synovial fibroblasts with IL‐22 in vitro suppressed their basal release of IL‐6, the production of this cytokine by cells from ES‐62–treated mice was still lower than that by cells from PBS‐treated mice with CIA. Collectively, these data indicate that ES‐62 may act via IL‐22 to suppress synovial fibroblast–mediated inflammation of the joints during established disease.

**Figure 6:**
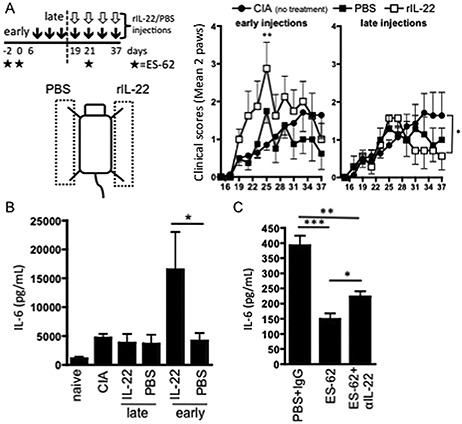
Recombinant IL‐22 (rIL‐22) modulates synovial fibroblast responses to IL‐17. **A,** Mice with CIA were injected twice weekly with rIL‐22 (0.25 μg/dose) in the right footpad or PBS (50 μl) in the left footpad (injection controls). Mice receiving no additional treatment were used as disease controls. IL‐22 injections were begun at the initiation phase of CIA (day 7 [early]) (n = 8) or around the time of onset of joint pathology (day 19 [late]) (n = 7). Values are the mean ± SEM (calculated using the mean clinical score of the 2 paws undergoing the same treatment in each individual mouse). **B** and **C,** Paws from individual mice with CIA receiving the indicated treatments, naive mice, and disease control mice with CIA were pooled to generate synovial fibroblast explant cultures representing the various treatment groups. Release of IL‐6 after 24 hours of treatment with medium alone (**C**) or with IL‐17 (**B**) was evaluated by enzyme‐linked immunosorbent assay. Values are the mean ± SEM (calculated using the mean of triplicate determinations of IL‐6 values from 3 independent cultures of cells from each treatment group). ∗ = *P* < 0.05; ∗∗ = *P* < 0.01; ∗∗∗ = *P* < 0.001 (*P* value shown for day 25 in the middle panel of **A** is versus untreated mice with CIA). See Figure 1 for other definitions.

To directly investigate the potential dual roles of IL‐22 in joint inflammation, mice with CIA were administered rIL‐22 in the right footpads (PBS in the left footpads) twice weekly from day 7 in order to mimic local up‐regulation of IL‐22 during both the initiation and the effector phases of disease (Figure 6). Treatment with rIL‐22 initially increased the articular score (peak on day 25) before beginning to mediate some resolution of joint inflammation, resulting in suppression of the articular score relative to that in untreated mice with CIA by the end of the experimental period. These protective effects were more pronounced when rIL‐22 was first administered to the footpads around the time of disease onset (twice weekly from day 19) to mimic the elevated levels observed in mice with established CIA that had been exposed to ES‐62 in vivo. With this protocol there was significant reduction in pathology (Figure 6A). Although the inflammation in the footpads that received this “therapeutic” administration of rIL‐22 was also reduced relative to that in PBS‐treated footpads of the same animals, it was evident that the pattern of IL‐22 first promoting and then resolving joint inflammation was paralleled, albeit to a lesser extent and following a slight delay, in the PBS‐treated joints. This suggests that the effects of rIL‐22 were being transferred to these limbs, presumably via systemic effects of the injected rIL‐22 or perhaps by its functional suppression of “pathogenic” fibroblasts, which has been reported to mediate clinical spreading of arthritis between joints (35).

Finally, to confirm that these pathogenic and protective effects of rIL‐22 were targeting synovial fibroblasts in the joint, we analyzed IL‐6 production by synovial fibroblast explant cultures from paws treated with rIL‐22 and from PBS‐treated control paws of the same animals. In accordance with their pathogenic status (35), synovial fibroblasts from mice with CIA exhibited enhanced IL‐6 production relative to those from naive mice (Figure 6B), and administration of rIL‐22 to the paw from the time of the initiation phase of CIA resulted in even higher production of IL‐6 by synovial fibroblasts. In contrast, when rIL‐22 administration did not begin until around the time of onset of joint pathology, the capacity to produce IL‐6 tended to be reduced toward the levels observed in naive mice (Figure 6B). Importantly, while synovial fibroblasts from ES‐62–treated mice exhibited reduced IL‐6 responses relative to those from mice treated with PBS (Figure 5C) or PBS plus IgG (Figure 6C), such desensitization was partially overcome in synovial fibroblasts derived from mice with CIA that were exposed to both ES‐62 and neutralizing anti–IL‐22 antibodies in vivo (Figure 6C), corroborating the antiinflammatory role of IL‐22 in ES‐62–mediated protection against joint inflammation.

## DISCUSSION

ES‐62 protects against CIA by targeting priming of a complex IL‐17–producing cellular network that involves dendritic cells and γ/δ and CD4+ T cells, and also by acting directly on Th17 cells (11). In this study, we investigated whether ES‐62–mediated suppression of IL‐22 responses also contributed to its protective effects in CIA, as this Th17‐associated cytokine has similarly been implicated in CIA pathogenesis (22, 23). Although we confirmed the pathogenic role of IL‐22 in the initiation phase of CIA, we found, surprisingly, that ES‐62 treatment appeared to enhance IL‐22 responses following onset of disease. Moreover, during established disease, serum levels of IL‐22 correlated inversely with articular scores, and local administration of IL‐22 reduced joint inflammation. Furthermore, ES‐62–mediated protection against CIA could be blocked by administration of neutralizing anti–IL‐22 antibodies (from day 19). Collectively, these data suggest that IL‐22 has dual pro‐ and antiinflammatory roles in CIA, with early, systemic IL‐17 and IL‐22 (Th22) responses cooperating to drive pathogenesis, while later, IL‐22 acts at the site of inflammation to counterregulate IL‐17 proinflammatory signaling and promote resolution of joint disease.

IL‐22 is involved in the host response to infectious diseases by promoting inflammation (36, 37) but, reflecting its tissue repair properties, it exerts both pro‐ and antiinflammatory actions in allergic and autoimmune inflammatory disorders (13, 19, 20, 38, 39, 40). Indeed, our results demonstrating dual pathogenic and inflammation‐resolving roles of IL‐22 are reminiscent of the findings in studies using models of ovalbumin‐induced airway hyperresponsiveness, in which IL‐22 appears to be essential for antigen sensitization (41) yet acts to resolve established airway inflammation. Likewise, in asthma patients, while serum IL‐22 levels are elevated and correlate positively with disease severity (41, 42), levels in bronchoalveolar lavage fluid correlate inversely with those of proinflammatory chemokines, and IL‐22 can inhibit the release of proinflammatory mediators by human bronchial epithelial cells (39). Results of other recent studies have called into question the idea that IL‐22 has a solely pathogenic role in arthritis, as antigen (methylated bovine serum albumin [BSA])–induced, IL‐17–mediated joint inflammation was found to occur independent of IL‐22 (43) and systemic administration of rIL‐22 was protective in late stages of disease in the CIA model (44). Further supporting the notion of an inflammation‐resolving role of this cytokine, IL‐22 has recently been shown to potentially modulate the IL‐23/IL‐17 inflammatory axis in RA patients by down‐regulating IL‐23 and IL‐17RC expression in fibroblast‐like synoviocytes (32).

Although full understanding of the mechanisms underlying the IL‐22–driven resolution of articular inflammation requires further investigation, the above findings are consistent with the notion that ES‐62 resets the balance of IL‐22/IL‐17 signaling in the inflamed synovium from proinflammatory toward desensitization of “pathogenic” synovial fibroblast responses, consequently reducing infiltration of effector cells and joint damage. Thus, and consistent with reports that IL‐22 promotes osteoclast differentiation from human monocytes via RANKL production by synovial fibroblasts in vitro (27), administration of rIL‐22 to the paws during the initiation phase of CIA resulted in enhanced basal and IL‐17–stimulated IL‐6 responses by synovial fibroblasts. In contrast, exposure to IL‐22 in vitro was found to suppress the levels of IL‐6 secreted by synovial fibroblasts derived from mice with established CIA, while synovial fibroblasts from ES‐62–treated mice with CIA, which exhibited elevated serum and joint levels of IL‐22 following disease onset, demonstrated desensitized basal and IL‐17–stimulated IL‐6 responses. These latter data are consistent with reports that Th17 cells from IL‐22–deficient mice induced synovial cells to produce higher levels of IL‐6 than those from wild‐type mice (23), suggesting that (aberrant) release of this proinflammatory cytokine may normally be limited by IL‐22. Our finding that neutralizing anti–IL‐22 antibodies could prevent both ES‐62–mediated desensitization of synovial fibroblast responses and protection against CIA strongly suggests that IL‐22 has a role in desensitizing synovial fibroblasts and promoting resolution of joint inflammation in established arthritis.

A key question therefore relates to the trigger that switches IL‐22 from a pro‐ to an antiinflammatory cytokine in CIA. Our data suggest that this occurs around the time of onset of joint pathology (days 20–25) as in the early stages, both systemically and locally in the joint, IL‐17 and IL‐22 (in the relatively low levels at which they are present) appear to act cooperatively (and may indeed both be derived from Th17 cells as described for IL‐1–driven arthritis [45]) to promote pathogenesis, whereas in the IL‐22–mediated protection phase, IL‐17 levels are high and IL‐17 and IL‐22 appear to be produced by antagonistic cell populations. It is not clear what are the major cell producers of “antiinflammatory IL‐22” or their precise targets other than synovial fibroblasts; elucidation of this may help explain the failure of neutralizing anti–IL‐22 antibodies to exacerbate disease when administered systemically following the onset of pathology. Moreover, in addition to reducing the levels of neutrophils infiltrating the joints, it appears that ES‐62 may have modified such cells functionally to a “protective IL‐22–producing phenotype,” perhaps suggesting that the parasite product provides additional signals to rewire cells to become “protective” sources of IL‐22 and/or targets of the antiinflammatory action of IL‐22.

It is therefore of interest that infection with the helminth *Trichuris trichiura* has been reported to therapeutically alleviate ulcerative colitis (46) by a mechanism that is dependent on expansion of IL‐22–positive cells. This suggests that induction of the tissue repair properties of IL‐22 could be a mechanism evolved by worms to promote healing of wounds arising from their invasion, to prevent harmful pathology to the host and/or inflammatory responses that could result in their expulsion. Exploiting the ability of helminth products such as ES‐62 to induce such inflammation‐ and wound‐resolving responses to treat autoimmune disorders is therefore an attractive prospect. Toward this end, we have obtained preliminary data suggesting that PC (the active moiety of ES‐62), when conjugated to BSA, also significantly promotes generation of joint inflammation–resolving IL‐22. Indeed, studies of PC–BSA were the starting point for our development of drug‐like derivatives that mimic the ability of ES‐62 to suppress CIA by targeting pathogenic IL‐17 responses (47) and provide proof‐of‐concept that exploiting the actions of helminth‐derived immunomodulators may potentially open novel avenues for drug discovery in RA.

## AUTHOR CONTRIBUTIONS

All authors were involved in drafting the article or revising it critically for important intellectual content, and all authors approved the final version to be published. Dr. M. Harnett had full access to all of the data in the study and takes responsibility for the integrity of the data and the accuracy of the data analysis.

**Study conception and design.** Pineda, W. Harnett, M. Harnett.

**Acquisition of data.** Pineda, Rodgers, Al‐Riyami.

**Analysis and interpretation of data.** Pineda, Rodgers, Al‐Riyami, W. Harnett, M. Harnett.
